# Robot-Assisted Radical Cystectomy with Modified Vesica Ileale Padovana (VIP) Neobladder Configuration Using a Hybrid Approach: Initial Experience

**DOI:** 10.3390/jpm13050802

**Published:** 2023-05-07

**Authors:** Fumitaka Shimizu, Satoru Muto, Kosuke Kitamura, Toshiyuki China, Tomoya Shirakawa, Tomoki Kimura, Takeshi Ieda, Masayoshi Nagata, Shuji Isotani, Yuki Nakagawa, Shigeo Horie

**Affiliations:** Department of Urology, Juntendo University Graduate School of Medicine, Tokyo 113-8431, Japankkitamu@juntendo.ac.jp (K.K.); tchina@juntendo.ac.jp (T.C.); t-shirakawa@juntendo.ac.jp (T.S.); to-kimura@juntendo.ac.jp (T.K.); tieda@juntendo.ac.jp (T.I.); m-nagata@juntendo.ac.jp (M.N.); shujiisotani@gmail.com (S.I.); y.nakagawa.zp@juntendo.ac.jp (Y.N.)

**Keywords:** bladder cancer, robot-assisted radical cystectomy, modified VIP neobladder, hybrid approach

## Abstract

Purpose: We developed a new technique to fold a neobladder (NB) simply by using a modified Vesica Ileale Padovana (VIP) with a hybrid approach. We provide a step-by-step description of our technique as it was used in this initial experience. Methods: A total of 10 male patients with a median age of 66 years underwent robot-assisted radical cystectomy (RARC) with an orthotopic NB via a hybrid approach from March 2022 to February 2023. After the isolation of the bladder and bilateral pelvic lymphadenectomy, Wallace plate creation was performed, and the robot was undocked. We extracorporeally performed the removal of the specimen and a side-to-side ileoileal anastomosis, and then the VIP NB posterior plate was rotated 90 degrees counterclockwise using a 45 cm detubularized ileum. The robot was redocked; then, circumferential urethra–ileal anastomosis, side-to-middle anterior wall closure, and ureteric afferent limb anastomosis were performed. Results: The median estimated blood loss was 524 mL, and the mean operative time was 496 min. Patients had a high continence rate, and no high-grade complications were observed. Conclusion: The NB configuration using the modified VIP method for a hybrid approach is a feasible surgical technique to minimize the movement of robotic forceps. In particular, it may be more useful in Asian individuals with narrow pelvises.

## 1. Introduction

In current RC practice, urinary diversion (UD) is roughly divided into cutaneous ureterostomy, ileal conduits, and orthotopic ileal NBs. An NB does not require a stoma and provides a high quality of life [1,2].

In 1954, Pyrah et al. performed the first-ever urethra–ileal anastomosis after total cystectomy [3]. At the time, since the ileum was sutured without detubularization, challenges such as urinary incontinence due to intestinal peristalsis and difficulty urinating were noted. Thereafter, it was found that NB reconstruction with detubularization of the ileum resulted in a high capacity and low pressure, leading to the development of many surgical procedures. Importantly, a lower pressure is maintained by using the ileum rather than the sigmoid colon. The currently used ileal NB reconstruction technique has become the standard. When an ileum of the same length, at approximately 35 cm or longer, is used, a W pouch or spheroid shape has a higher capacity than a U pouch and tube [4]. According to Laplace’s law, a spherical pouch allows the highest capacity and lowest pressure. Studer showed that folding the U-shaped ileum enabled the NB to achieve a low pressure and high capacity [5]. Large reservoirs may increase residual urine, leading to the need for self-catheterization. The reservoirs do grow larger over time. Hence, it is essential to aim for a spherical, low-pressure NB reconstruction via detubularization and double folding using an ileum with an appropriate length. Studer et al. reported that when a 40 cm segment of ileum was used to create an NB, the postoperative capacity was approximately 150 mL, which increased to around 500 mL over time [5]. Hautmann stated that the ultimate goal of UD is not only to provide a means of urinary outflow and protection of the upper urinary tract, but also continence and the ability to urinate voluntarily [6]. Recently, many surgeries have shifted from open and laparoscopic surgery to robotic surgery, which has reached its full development [7]. NB reconstruction benefits greatly from robotic surgery, and there are various intracorporeal reconstruction methods.

The surgical steps for robotic NB configuration are different from the procedure of open radical cystectomy (ORC), even if the reconstructive method is the same. For example, the shape of the NB may change depending on whether a urethral anastomosis is performed before or after folding.

Before detubularization, the ileum may not reach the urethra due to mesenteric tension. In such cases, the mesentery is dissected and detubularized to facilitate the mobilization of the ileum to the urethra.

Asian individuals with narrow pelvises may have poor urethral visibility with intracorporeal neobladders (ICNBs). The visibility of the urethra may be improved by cutting the mesentery and detubularization first, creating only the posterior wall of the NB, and then performing the circumferential anastomosis between the posterior wall of the NB and urethra. In Asian individuals with narrow pelvises, the robot forceps tend to hit the pelvic wall. In addition, it would be better if the folding of the NB could be performed tension-free from the side to the center so that the movement of the robotic forceps is minimized. We considered the alteration of the posterior wall of the NB to allow such robotic forceps movements.

VIP has a 30-year-old history as a spherical NB capable of achieving low pressure [8]. We developed a new technique to fold NBs simply by using a modified VIP NB via a hybrid technique. This technique may be useful for Asian individuals with narrow pelvises. We provide a step-by-step description of our technique.

## 2. Material and Methods

### 2.1. Patients

Bladder cancer was confirmed pathologically in all patients by transurethral resection of bladder tumor (TUR-Bt) prior to RARC. Between March 2022 and February 2023, 10 patients underwent RARC with a hybrid NB for bladder cancer. RARC was performed with a six-port transperitoneal approach using the daVinci Xi system (Intuitive Surgical G.K., Tokyo, Japan) in the 26-degree Trendelenburg position. After RARC, bilateral pelvic lymphadenectomy was performed on all the patients. We present the surgical steps of robot-assisted hybrid NB reconstruction after pelvic lymphadenectomy. All perioperative complications were classified according to the Clavien–Dindo classification (I: complications needing only bedside intervention via oral medications; II: complications needing only intravenous medications, total parenteral nutrition, enteral nutrition, or blood transfusion; III: complications needing interventional radiology, therapeutic endoscopy, intubation, angiography, or operation; IV: complications causing residual and lasting disability requiring major rehabilitation of organ resection; V: complications causing death).

### 2.2. The Hybrid Approach for the NB Reconstruction

In the hybrid approach, an NB is created combining the advantages of an extracorporeal neobladder (ECNB) and an ICNB through an infraumbilical midline incision. For women, it is possible to externalize a specimen through the vagina. Therefore, women do not require additional skin incisions like men. However, in the case of men, an incision is always required for removal of the specimen, so an incision can be made to manipulate the ileum extracorporeally. The surgical procedure performed after bilateral pelvic lymphadenectomy is described below.

### 2.3. Creation of a Wallace Plate

The left ureter was guided to the right side posterior to the sigmoid colon. The bilateral ureteric ends were spatulated and a Wallace plate was made using continuous 13 cm 4-0 polydioxanone sutures. Running sutures were performed from the proximal side to the distal side. We fixed the Wallace plate to the abdominal wall intracorporeally.

### 2.4. Mobilization of the Ileum

**Figure 1:**
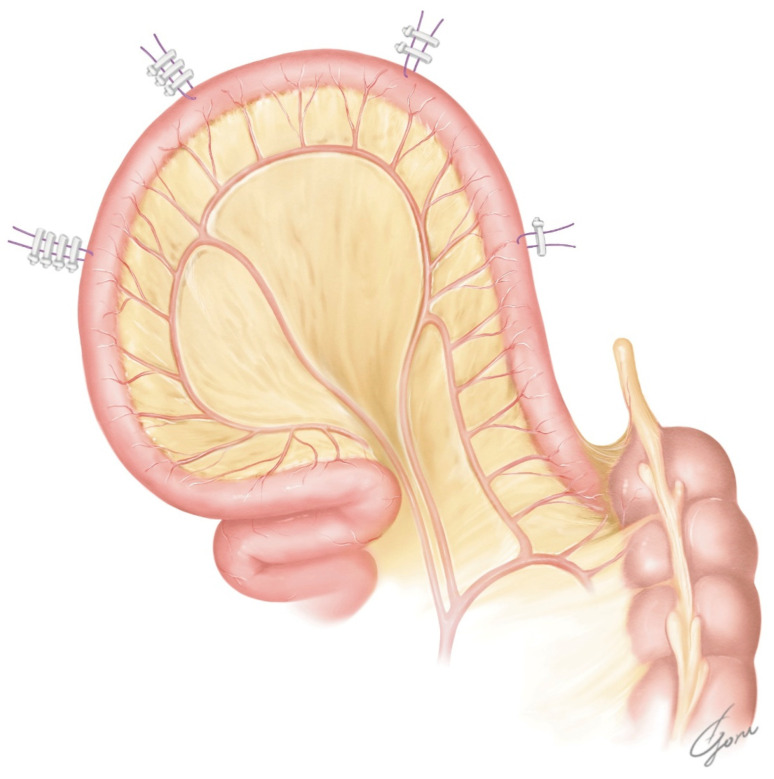
Marking the ileal turning point for the neobladder. The first clip is 20 cm from the terminal ileum, the second clip is 15 cm from first clip, the third clip is 15 cm from the second clip, and the fourth clip is 15 cm from the third clip.

Prior to extracorporeal manipulation, the ileal turning points for the NB were marked while attaching the ileum to the pelvic floor (Figure 1). We used the clips as shown in the figure to indicate the location of the ileum. Distance was measured using a 10 cm suture. First, we marked a point 20 cm from the ileocecal region, and then marked three points 15 cm apart toward the oral side from that point. In this operation, we approached the next point to be marked while still holding the previous suture with the fourth arm. If the central point between the second and third clips reaches the pelvic floor, a urethral anastomosis does not present a problem. This procedure prevents the misjudgment of the distance to the urethra for urethra–ileal anastomosis.

### 2.5. Extracorporeal Manipulation and Creation of the Posterior Plate

**Figure 2:**
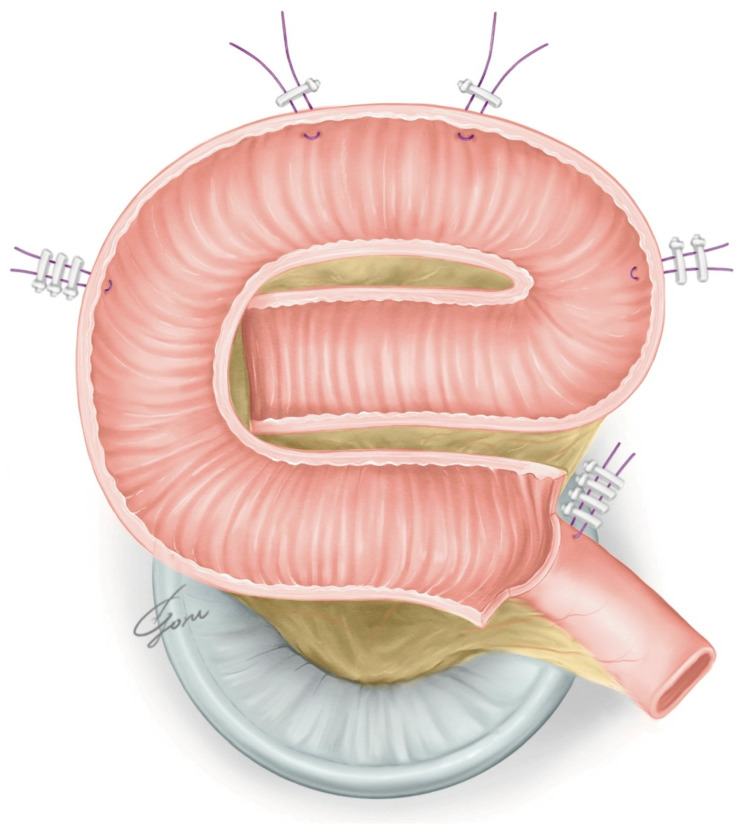
The design of our posterior plate using the modified VIP method. Two supporting sutures were placed on the ileum near the urethra–ileal anastomosis.

After undocking the robot, an infraumbilical midline incision of approximately 7 cm was placed, and the bladder specimen was removed. An Alexis wound retractor (Medical Leaders, Tokyo, Japan) was placed and the ileum was dissected extracorporeally according to the markings. Then, a side-to-side ileoileal anastomosis was performed using a 60 mm EndoGIA stapler (Covidien, Medtronic, Tokyo, Japan). Approximately 45 cm of ileum was then detubularized, leaving 10 cm of the afferent limb. The ileum near the urethra–ileal anastomosis was incised slightly closer to the mesentery. We placed two supporting sutures with a clip on the ileum near the urethra–ileal anastomosis for traction. Figure 2 shows the design of our posterior plate, which is ovular. It has a reverse spiral shape opposite to that of the original VIP method to allow it to move the afferent limb forward. Additionally, the posterior wall of the original VIP was rotated 90 degrees counterclockwise to allow the NB to be folded from the side to the center. When the formation of the posterior plate of the NB was completed, it was checked again to determine whether the posterior plate could be moved to the level of the pubic bone. If mobilization of the posterior plate was still difficult at this point, additional dissection of the mesentery or a relaxing incision of the mesentery was considered. With the 6Fr ureteral stent passing through the afferent limb, the posterior plate was returned to the intracorporeal. The Alexis wound retractor was capped.

### 2.6. The Urethra–Ileal Anastomosis and Anterior Wall Closure

**Figure 3:**
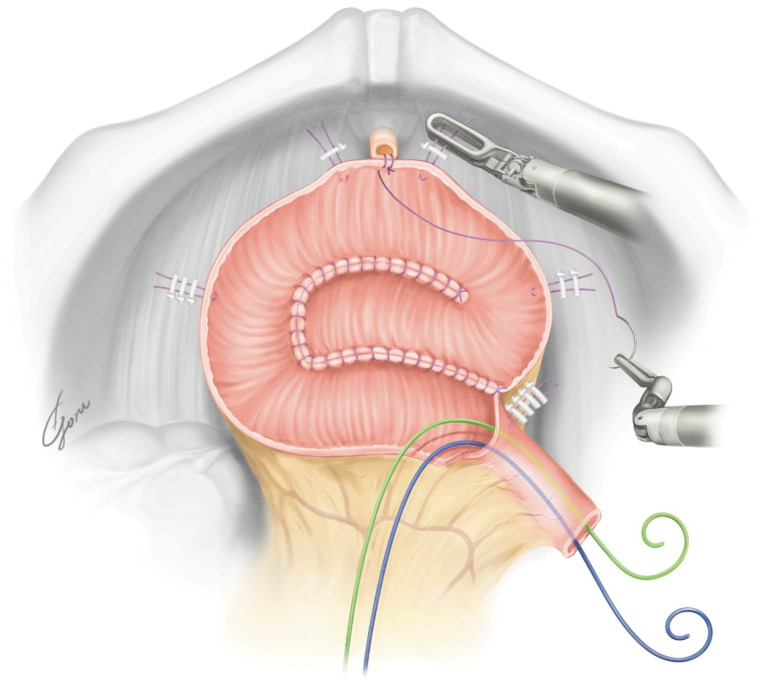
The shape of the posterior plate when pulled toward the urethra.

**Figure 4:**
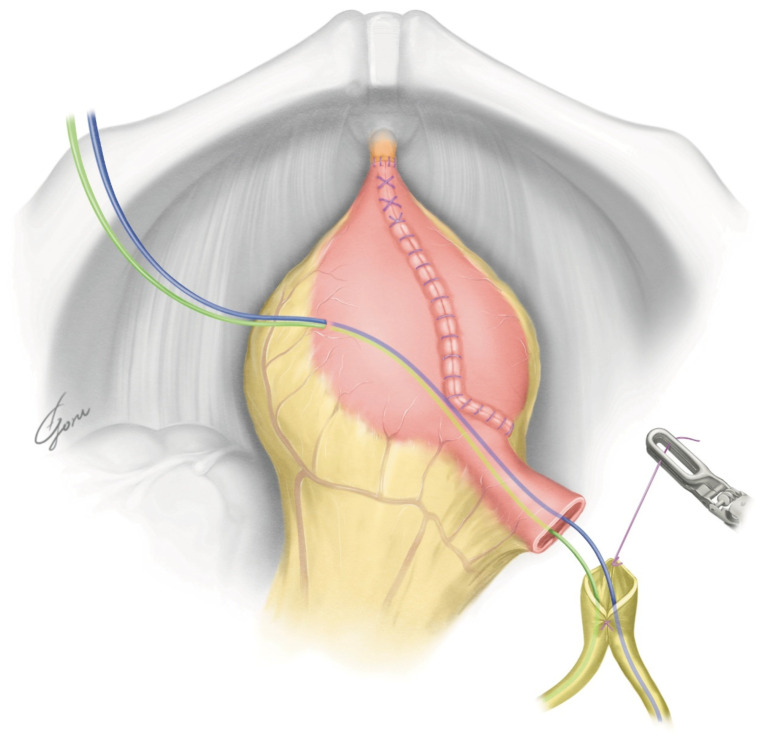
The schema of the NB created using our method.

The robot was redocked head-down at 10–12 degrees. The right supporting suture of the posterior plate was grasped with 4th arm forceps and the posterior plate was mobilized to the pelvic floor. A circumferential urethra–ileal anastomosis was performed using 3-0 VLoc sutures, similar to a robot-assisted radical prostatectomy (RARP) (Figure 3). The advantage of performing a circumferential anastomosis after creating a posterior plate is the visibility of the urethra during an anastomosis. If an opening is made and a urethral anastomosis is performed before detubularization, the part to be anastomosed may be difficult to visualize in Asian individuals with narrow pelvises. The anterior wall of the NB was continuously sutured so it was water-tight and tension-free using 3-0 VLoc sutures from the left and right toward the center. Via this manipulation, a folded structure could be obtained. A 20Fr Foley catheter was inserted and inflated with 20cc water when the anterior wall was half-closed. A bilateral 6Fr ureteral stent was passed through the anterior wall into the abdominal wall and inserted into each ureter using a guidewire. The remaining anterior wall was closured. Figure 4 shows the schema of the ileal NB created via our method.

### 2.7. The Ureteric Afferent Limb Anastomosis

The clip attached to the ureter was moved toward the foot using the 4th arm forceps. A running suture of the afferent limb and Wallace plate was performed using continuous 4-0 polydioxanone sutures bilaterally. Running suture was carried out from the proximal side to the distal side. The NB was irrigated by saline to ensure a water-tight closure. A passive tube drain was placed in the pelvis through the port site, and the robot was undocked.

### 2.8. Postoperative Care

All patients were managed on a clinical care pathway postoperatively. We recommend clear liquid on postoperative day (POD) 1, and diet is advanced with return of bowel function. Early ambulation was instituted in all patients. We started to irrigate the NB through a urethral catheter from around POD 3. The abdominal drain was removed when the output was <150 mL/d, and fluid biochemistry excluded urine. Ureteral catheters were removed one by one approximately one week after surgery. If there was no leakage on cystography two weeks after surgery, the Foley catheter was removed. The patients were instructed to urinate every two or three hours.

## 3. Results

**Figure 5:**
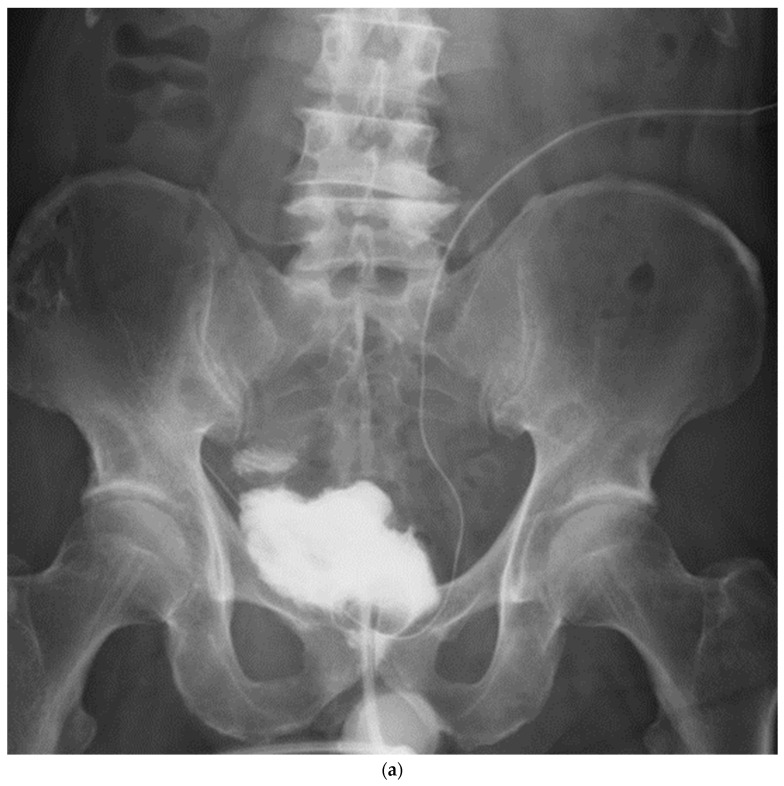

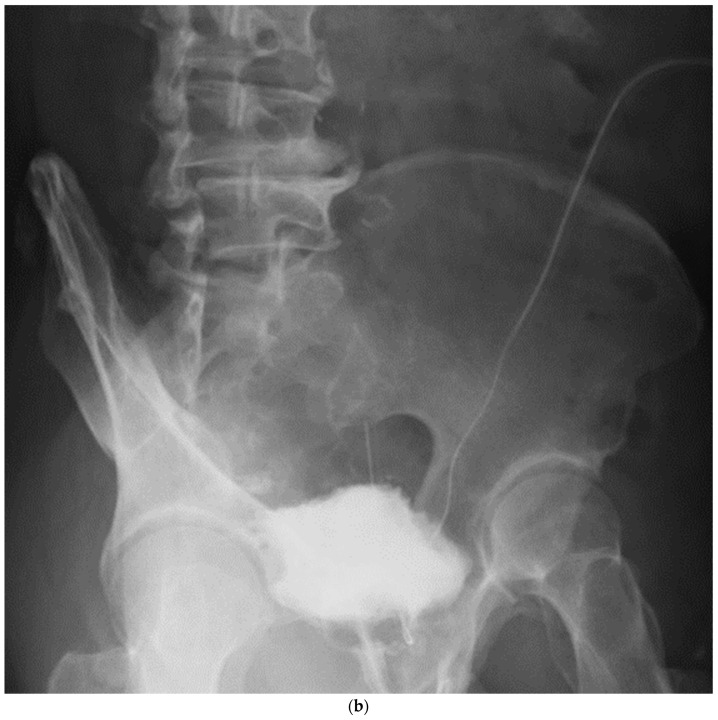
Cystography at 2 weeks after surgery. (**a**). Frontal view, (**b**). Oblique view.

Patient demographics and variables are shown in Table 1. Most patients received neoadjuvant chemotherapy, and pT0 was achieved in two (20%) patients. Pathology confirmed organ-confined disease in seven (70%) patients and locally advanced disease in three (30%) patients. Nerve-sparing surgery was performed in three cases. Total operative time was 496 (418–815) min, and mean estimated blood loss was 524 (110–850) mL. No patients received intraoperative blood transfusions. In one patient, the ureteral stent was dislodged immediately after surgery, but no urine leakage was noted thereafter without reinserting. There were nine minor complications, as shown in Table 2. One patient had subileus but improved conservatively. One patient had urine leakage at the urethral anastomosis and a prolonged drain. Diarrhea was observed in two patients and improved with an oral antiflatulent agent. Vitamin B12 deficiency was observed in one patient, which was improved by oral supplementation. The urinary tract infection in one patient improved with an intravenous drip of antibiotics. No readmissions due to complications were observed. Continence was defined as zero or one safety pad, and postoperative early daytime continence was achieved in nine (90%) patients. Early nocturnal continence was achieved in eight (80%) patients. The three patients who underwent nerve-sparing RC were continent both during the daytime and nighttime. No patients required clean intermittent catheterization. There was no uretero-afferent limb stricture, urethro-enteric stricture, or decline in renal function. Figure 5 shows the cystography of the modified VIP NB two weeks after surgery.

It maintained the spherical shape established using our technique.

## 4. Discussion

In robot-assisted surgery, the robotic arm can move freely, making it possible to perform suturing that was previously difficult with laparoscopic surgery. However, if the pelvis is narrow, the robotic forceps hit the pelvic wall. We developed a new technique for folding the pouch conveniently by using a modified VIP NB. The modified VIP, which minimizes the movement of the robotic forceps, is beneficial in Asian individuals with narrow pelvises. A hybrid approach that takes advantage of features of both intracorporeal urinary diversion (ICUD) and extracorporeal urinary diversion (ECUD) is a useful technique. Masumori et al. reported a hybrid approach for Studer NB reconstruction [9]. Iwamoto et al. also reported a hybrid approach in RARC [10]. The hybrid approach allows effective use of the incision to remove the bladder and can shorten head-down time. In this approach, the ureteral stent can be passed extracorporeally through the afferent limb and back into the abdominal cavity without robotic forceps passing through the afferent limb. ICUD may utilize indocyanine green (ICG) to assess mesenteric blood flow. A hybrid approach can be assessed mesenteric arterial pulsation directly. Additional resection of the mesentery can be carried out in situ if movement of the posterior wall of the NB attached to the pelvic floor is poor. On the other hand, additional time is required to redock the robot.

In ICNB reconstruction, establishing UD after RARC is considered the most challenging step of the entire surgical procedure. Despite the increasing precedence of RARC, the majority of centers perform ECNB reconstruction because of the perceived difficulty of bowel reconfiguration and justified concerns regarding time efficiency compared with the open approach. Recently, the number of ICUDs performed has increased rapidly in NB reconstraction, along with the evolution of devices [11]. In Japan, the Studer or the modified Studer was often selected for open NB reconstruction. The Studer NB is still commonly the preferred technique despite a shift from ORC to RARC. Otaola-Arca et al. reported that Studer reconstruction was selected in 70% of ICNB surgeries [12]. In RARC, the NB reconstruction steps used in the open approach need to be modified. Proper intestinal positioning and manipulation are essential in ICNB reconstruction. The Ligaloop bands technique by Jonsson et al. is a popular choice [13]. An opening is created contralateral to the mesentery, and a urethra–ileal anastomosis is performed. Subsequently, a 60 mm stapler is inserted through the left port to transect the ileum and re-establish it with a side-to-side ileoileal anastomosis. Some surgeons use a suprapubic port to avoid excessive mobilization and bowel rotation against the stapler. Alternatively, after fixing the ileum to the pelvic floor, the intestine is separated, the transected bowel segment is detubularized, and a posterior plate is created. Thereafter, an opening is created and anastomosed with the urethra and posterior plate. Alternatively, a circumferential urethra–ileal anastomosis is performed without creating an opening. To extract the specimen, the Pfannenstiel incision is often carried out. The Karolinska-modified Studer, USC-modified Studer, Hautmann, Pyramid, Y-pouch, and VIP NB have been reported as typical ICNB reconstruction methods [14]. The Karolinska-modified Studer approach is the method most surgeons use to perform this procedure. It results in high daytime and nighttime continence rates [15,16]. The USC-modified Studer technique enables easy folding in robotic surgery via rotating the posterior plate 90 degrees counterclockwise [17]. However, in this technique, the posterior wall of the NB is pulled toward the urethra during a urethra–enteric anastomosis. Hence, when the anterior wall is laterally sutured, the NB could end up being vertically elongated. Subsequently, they updated the technique to include an additional fold to establish a more spherical shape [18]. In our modified VIP NB procedure, the shape of the posterior plate does not change significantly even when it is pulled toward the urethra (Figure 3). Our method achieves simple robotic manipulation without losing the folded structure. The advantages of an ICNB include a reliable urethra–enteric anastomosis, low insensible perspiration, and no need for excessive dissection of the ureter. These advantages also apply to the hybrid approach. If UD is performed extracorporeally, the ureteral length is typically longer than that achieved in intracorporeal UD, possibly contributing to a higher uretero-enteric stenosis rate [19]. The disadvantages include the extension of the operative time and head-down time. However, recent reports show that the total operative time is similar to that of ECNB reconstruction [11]. Although the use of an ICNB has been steadily increasing over time, it is associated with a five-times-higher risk of rehospitalization than an ECNB, which necessitates due circumspection [11]. Longer operative times and lower annual numbers of RARC procedures performed are risk factors for high-grade complications and readmission. ICNB reconstruction should be performed at a high-volume center.

Table 3 shows the reconstruction techniques for ICNB reconstruction and the hybrid approach [9,13,17,18,20,21,22,23,24,25,26,27,28,29,30,31,32,33,34,35,36]. We also demonstrate the direction in which the posterior plate is folded intracorporeally. The goal of NB reconstruction is to create a spherical, low-pressure reservoir with an appropriate intestinal length. A properly compliant NB prevents urinary incontinence. It also prevents ureter reflux and protects renal function. While there are various NB reconstruction methods, which method is used may not be functionally significant as long as the folded structure is firm. However, care should be taken in modifying the steps of NB reconstruction from ORC to RARC. In the past, longer detubularization tubes were utilized to create a large NB, but 40–45 cm detubularization tubes have recently become mainstream because NBs grow over time. In a surgical procedure that uses the afferent limb, the length of the afferent limb is often 10–15 cm. We used a 45 cm detubularization tube and a 10 cm afferent limb. In the urethra–enteric anastomosis part, an opening is often created at the antimesenteric site of the ileum before detubularization, and a urethral anastomosis is performed [13,20,21,22,23,24,26,27,31,32,35,36]. Alternatively, a circumferential urethra–ileal anastomosis is performed after detubularization [9,17,18], or an opening is created after detubularization, and a urethral anastomosis is performed [28,33]. In ORC, it is common to create a pouch and then perform urethral anastomosis, unlike in RARC. If the opening is made intracorporeally in the ileum before detubularization and the urethra is anastomosed and a pouch created, the urethra may be displaced and bent. A bent urethra prevents urination and prohibits urinary catheterization. Simone et al. performed NB reconstruction without detubularization of the NB neck to enable a physiological urethral anastomosis [34]. A circumferential urethra–ileal anastomosis is more natural because the NB neck is formed with the urethra as the fulcrum. To the best of our knowledge, an evaluation of varying urethral stricture rates and residual urine volumes due to differences in the urethral anastomosis has not been carried out. This is a topic for future studies. The direction in which the NB is folded is a challenging decision in robotic surgery. For the Studer NB, in open surgery, the NB is often folded from the bottom, which is the lowest point of the ileum mesentery, to the top or obliquely upward. In robotic surgery, some techniques fold the NB from top to bottom, and suturing must be performed at the lowest point of the mesentery. We improved the design of the posterior plate so that the NB can be folded from side to center. This procedure is preferable because the folding can be easily performed by robotic forceps and there is no tension when closing the NB anterior wall. We must be conscious of spherical NBs. In robotic NB reconstruction preceded by detubularization, a urethra–ileal anastomosis is often performed after posterior wall configuration. Therefore, it is necessary to consider the possibility that a large amount of tension is applied toward the foot during NB configuration under the Trendelenburg position, resulting in an NB shape that is different than expected. In our technique, the NB is created with this point in mind. We have a unique point of view that focuses on the fact that the shape of the NB changes when the surgical order for the NB configuration is changed, even if the ICNB reconstruction method is the same as that carried out in ORC. Many different NB shapes have been reported, and it is difficult to determine which method is better. It is important that the transition to robotic surgery does not compromise the quality of NB function. Ureteral anastomoses often use the Bricker or Wallace technique. Ureteral stents are inserted percutaneously or transurethrally. The Wallace technique requires guiding the left ureter to the right side in front of the sacral promontory. The Wallace technique is known to reduce the ureteral anastomosis stricture rate [37]. However, if the ureter is short, it may be better to anastomose the ureter directly into the NB.

The procedure for ECNB reconstruction generally utilizes an infraumbilical midline incision of approximately 7 cm [38]. This position is the optimal location for manipulating the ileum and the ureter. A wound retractor with a lid is often used. The ileum is mobilized outside the wound to perform an ileo-ileal anastomosis and to create an NB. The robot is redocked and a urethra–enteric anastomosis is performed. Finally, an anastomosis of the afferent limb and the ureter is performed. By performing an anastomosis of the ureter and the afferent limb after the urethra–ileal anastomosis, the afferent limb is immobilized, which minimizes any significant movement. This technique is relatively simple, and the learning curve is small. An NB can be created by multiple surgeons and can be easily folded, shortening the operation time. The head-down time can also be shortened, which may reduce complications. On the other hand, additional time might be needed in undocking to create an NB. The main disadvantage of this technique is that a urethra–enteric anastomosis cannot be performed first. In some cases, the NB may not reach the urethra, which increases repair difficulty. Yanagisawa et al. reported a technique for facilitating a urethro-ileal anastomosis laparoscopically by inserting an extracorporeally created NB and fixing it in the pelvic floor [39]. It is important to perform a simulation before an anastomosis to ensure that tension is not applied to the mesentery when the NB is folded and when the NB and urethra are anastomosed. By selecting and marking the ileum for the urethra–ileal anastomosis before starting the extracorporeal operation, it may be possible to prevent the NB from failing to reach the urethra.

Urinary incontinence remains a common postoperative complication and impairs patients’ QOL. In RARC, the continence rate of pouches constructed with a 40–50 cm detubularized ileum is high for all of the reconstruction methods [18]. Data from the ICUD-EAU International Consultation on Bladder Cancer indicate a daytime continence rate of 92% for men and 85% for women and a nighttime continence rate of 76% for men and 72% for women [40]. Continence is achieved when the outlet pressure exceeds the reservoir pressure. However, the nighttime continence rate is slightly reduced by intestinal water secretion and less outlet contraction [41]. Low-dose desmopressin has been reported to be effective for nocturia and nocturnal enuresis in patients with an NB [42]. Satkunasivam et al. reported that ICNBs are inferior in the use of pads between an open NB and ICNB [43]. This may be due to differences in the follow-up periods. Additionally, further research is needed to determine whether there are differences between the ICNB surgical techniques.

Few studies have evaluated urodynamics following ICNB reconstruction. Koie et al. reported an NB capacity of 285 mL, an NB pressure of 26.5 cm H_2_O, and a urethral closure pressure of 46.8 cmH_2_O at 12 months for a cross-folded U-shaped NB with a 40 cm ileum [28]. The USC group reported an abdominal leak point pressure of 40–50 cm H_2_O as determined via provocative testing. Di Maida et al. showed the superiority of ICNBs to open groups in terms of urgency domain, psychological status, and physical self-acceptance in FACT-BL and EORCT-QLQ-C30 [44]. Postoperative urodynamic assessment at 6 months showed significantly higher NB compliance in the ICNB group.

Nerve-sparing RC may contribute to the continence rate. Cheng et al. reported that nerve-sparing techniques might help patients achieve a relatively higher functional trifecta [45]. Furrer et al. reported the usefulness of seminal-vesicle-sparing RC in erectile function and daytime urinary continence at all points [46]. He et al. reported lateral-capsule-sparing and combined pelvic reconstruction techniques for improving early daytime and nighttime continence rates [47].

However, the possibility of incidental prostate cancer coexistence must be considered in prostate-capsule- and seminal-vesicle-sparing techniques.

Gastrointestinal complications occur with a certain probability in RC. Among them, ileus is the most common. Patients undergoing ICUD recontraction with RARC showed no difference in the frequency of gastrointestinal complications in RCTs compared with those undergoing ORC [48]. Compared with ECUD, patients undergoing ICUD have a lower rate of gastrointestinal complications, readmission at 30 days, and postoperative complications at 90 days [49]. Even when it is limited to NB patients, no increased gastrointestinal complications were seen in ICNB reconstruction compared to ECNB reconstruction in one study [11].

In our initial experience, there were only minor complications. A modified VIP NB using a hybrid approach may be considered a feasible technique. This study had a limitation. Although our experience provided a view of perioperative outcomes, long-term follow-up data are lacking. Anceschi et al. reported on the evaluation of the overall survival in the era of RARC [50]. We thus need to evaluate long-term functional outcomes and survival. We took a little longer on our operative time. It may be important to have some stability in the team of surgeons. In addition, real-time fluorescent confocal microscopy assessment of urethral and ureteral margins may also be useful to reduce operative time [51].

We need to make efforts to shorten the operative time in order to reduce the burden on the patient.

## 5. Conclusions

A hybrid approach that combines the advantages of both ICUD and ECUD is very useful. The modified VIP NB technique using a hybrid approach is a feasible method of minimizing robot arm motion in Asian individuals with narrow pelvises. This technique provides a satisfactory perioperative functional outcome. Further accumulation of long-term follow-up data is expected in the future.

## Figures and Tables

**Table 1:** Patient demographics and variables.

Variable	Value
Gender, *n* (%)	
Male	10 (100)
Female	0 (0)
Median age, year (range)	66 (60–70)
Clinical T stage, *n* (%)	
CIS	1 (10.0)
Ta	0 (0)
T1	1 (10.0)
T2	5 (50.0)
≥T3	3 (30.0)
Neoadjuvant chemotherapy use, *n* (%)	9 (90.0)
RC histology, *n* (%)	
pT0	2 (20.0)
CIS	1 (10.0)
pTa	0 (0)
pT1	2 (20.0)
pT2	2 (20.0)
≥pT3	3 (30.0)

**Table 2:** Short-term and long-term complications.

Complications	Incidence, *n* (%)
Short-term complications	
Surgical	
Neobladder leakage	1 (10)
Gastrointestinal	
Ileus	1 (10)
Diarrhea/vomiting	2 (20)
Vitamin B12 deficiency	1 (10)
Infection	
Urinary tract infection	1 (10)
Long-term complications	
Daytime incontinence	1 (10)
Nighttime incontinence	2 (20)
Readmission due to complications	0 (0)
Grade of Clavien system complications	
Minor (Grade I–II)	9
Major (≧Grade III)	0

**Table 3:** Detailed comparison of the robotic orthotopic ileal neobladder from literature review series.

Institution	University of Southern California, USA	Karolinska, Sweden	Ankara, Turkey	North Carolina University, USA	Eberhard-Karls University, Germany	Saint-Augustin, France	Hirosaki University, Japan	London University, UK	Queen Elizabeth II, Canada	Third Military Medical University, China	Rush University, USA	Padova University, Italy	Regina Elena, Italy	Korea University, Korea	Sapporo Medical University, Japan	Juntendo University, Japan
Author	Goh et al. [17] Chopra et al. [18]	Jonsson et al. [13], Tyritzis et al. [20], Collins et al. [21]	Schwentner et al. [22], canda et al. [23], Akubulut et al. [24]	Pruthi et al. [25]	Sim et al. [26]	Asimakopoulos et al. [27]	Koie et al. [28]	Tan et al. [29]	Butt et al. [30]	Zhou et al. [31]	Whelan et al. [32]	Dal Moro et al. [33]	Simone et al. [34]	Pyun et al. [35] Kang et al. [36]	Masumori et al. [9]	Our series
*n*	8	36 70 86	62 23 -	3	73	40	22	20	4	40	2	-	45	-	-	10
Length of ileum used, cm	60	50	50	N/A	40	40	40	50	65	40	45	45	42	60	55	55
Detubularization, cm	44	40	40	N/A	28	N/A	40	50	N/A	40	45	40	42	60	40	45
Afferent limb, cm	Yes, 11	Yes,10	Yes, 10	No	Yes, 6 × 2	No	No	No	Yes, N/A	No	No	No	No	No	Yes,15	Yes,10
Timing of the urethro-ileal anastomosis	After posterior plate reconstruction	Start of the reconstruction (before detuburiztion)	Start of the reconstruction (before detuburiztion)	After pouch completion	Start of the reconstruction (before detuburiztion)	Start of the reconstruction (before detuburiztion)	Start of the reconstruction (after detuburiztion)	After pouch completion	After pouch completion	Start of the reconstruction(before detuburiztion)	Start of the reconstruction (before detuburiztion)	Start of the reconstruction (after detuburiztion)	After posterior plate reconstruction	Start of the reconstruction (before detuburiztion)	After posterior plate reconstruction	After posterior plate reconstruction
Method of the urethro-ileal anastomosis	Circumferential	Opening	Opening	Opening	Opening	Opening	Opening	Opening	Opening	Opening	Opening	Opening	Opening	Opening	Circumferential	Circumferential
Uretero-enteric anastomosis	Bricker	Wallace	Wallace	Bricker	Bricker	Wallace	Split nipple technique	Bricker	Bricker	Bricker	Bricker	Bricker	Split nipple technique	Bricker	Bricker	Wallace
Direction to fold	Bilateral sides to center	Diagonally downward	Diagonally upward	N/A	Single folding, Bilateral sides to center	Top to bottom	Top to bottom	Up down left right to center	Bilateral sides to center	Bilateral sides to center	Top to bottom	Top to bottom	Top to bottom	Single folding, Bilateral sides to center	Diagonally upward	Bilateral sides to center
Ureteral stenting insertion	Percutaneous, internalized	Per urethra/Percutaneous, internalized/Percutaneous externalized	Percutaneous externalized/Per urethra	Per urethra	Percutaneous internalized	Per urethra	Percutaneous internalized	Percutaneous externalized	Percutaneous internalized	N/A	None	None	Percutaneous internalized	Percutaneous internalized	Percutaneous externalized	Percutaneous externalized/Percutaneous internalized
Shape	Modified Studer	Modified Studer	Modified Studer	U	Y	Y (similar to Cross-folded U)	Cross-folded U	Pyramid	Hautmann W	Hautmann W	Ves.Pa	Ves.Pa	Modified VIP	Camey	Modified Studer	Modified VIP
Operative time, min, (range or SD)	450 (420–780)	480 (330–760) 420 (265–760) -	476 (310–690) 594 -	318 (258–696)	442 (280–690)	315 (172–400)	430 (349–476)	150 (120–360)	523 (75)	320 (230–500)	543	-	305 (282–345)	649	-	496 (418–615)
Estimated blood loss, mL, (range or SD)	225 (100–700)	625 (200–2200) 500 (100–2200) -	385 (200–800) 430 -	221 (50–400)	347 (50–800)	395 (0–700)	300 (119–450)	260 (100–500)	238 (48)	300 (100–2000)	225	-	210 (50–250)	148	-	524 (110–850)

## Data Availability

Data are available upon reasonable request.
